# Evolutions in Commercial Meat Poultry Breeding

**DOI:** 10.3390/ani13193150

**Published:** 2023-10-09

**Authors:** Anne-Marie Neeteson, Santiago Avendaño, Alfons Koerhuis, Brendan Duggan, Eduardo Souza, James Mason, John Ralph, Paige Rohlf, Tim Burnside, Andreas Kranis, Richard Bailey

**Affiliations:** 1 Aviagen Group, Newbridge EH28 8SZ, UK; savendano@aviagen.com (S.A.); akoerhuis@aviagen.com (A.K.); tburnside@aviagen.com (T.B.); rbailey@aviagen.com (R.B.); 2 Aviagen Ltd., Newbridge EH28 8SZ, UK; bduggan@aviagen.com (B.D.); or andreas.kranis@roslin.ed.ac.uk (A.K.); 3 Aviagen Inc., Huntsville, AL 35805, USA; emsouza@aviagen.com (E.S.); jmason@aviagen.com (J.M.); 4 Aviagen Turkeys Ltd., Tattenhall CH3 9GA, UK; jralph@aviagen.com; 5 Aviagen Turkeys Inc., Lewisburg, WV 24901, USA; prohlf@aviagen.com; 6 The Roslin Institute, Royal (Dick) School of Veterinary Studies, Midlothian EH25 9RG, UK

**Keywords:** chickens and turkeys, balanced breeding, breeding goal, trait development, antagonism management, quantitative genetics, genomics, bird welfare and health, robustness, diversity

## Abstract

**Simple Summary:**

Poultry meat is an affordable, lean source of animal protein. Where does it come from? Broilers (meat chickens) or turkeys raised on farms stem from core breeding families, which are crossed to generate commercial hybrids. It takes around four years for the birds from the core families to the birds on the farms. Over time, poultry breeding has developed from primarily looking at production (e.g., live weight and egg production) to balanced, holistic breeding, including a wide range of attributes (e.g., gait, leg health, robustness and cardiovascular health). All birds in the core breeding families are carefully measured for over forty items covering bird health and welfare, robustness, environmental impact, reproduction and production. Modern poultry breeding aims for the holistic improvement of all the traits included in a broad and balanced breeding goal. New and improved selection techniques and analytical tools are continuously developed to allow increases in the accuracy of selection’s long-term progress. There are different broiler and turkey genotypes for different market requirements, and commercial portfolios will continue evolving with future markets and customer preferences.

**Abstract:**

This paper provides a comprehensive overview of the history of commercial poultry breeding, from domestication to the development of science and commercial breeding structures. The development of breeding goals over time, from mainly focusing on production to broad goals, including bird welfare and health, robustness, environmental impact, biological efficiency and reproduction, is detailed. The paper outlines current breeding goals, including traits (e.g., on foot and leg health, contact dermatitis, gait, cardiovascular health, robustness and livability), recording techniques, their genetic basis and how trait these antagonisms, for example, between welfare and production, are managed. Novel areas like genomic selection and gut health research and their current and potential impact on breeding are highlighted. The environmental impact differences of various genotypes are explained. A future outlook shows that balanced, holistic breeding will continue to enable affordable lean animal protein to feed the world, with a focus on the welfare of the birds and a diversity of choice for the various preferences and cultures across the world.

During the last half century, the genetic improvement in broiler chicken and turkey breeds has resulted in good health, welfare and sustainability alongside increased productivity and robustness across a wide range of production conditions worldwide. This paper reviews the key developments from domestication to modern commercial primary poultry breeding. It aims to provide a concise overview of the developments and progress made in commercial meat chicken and turkey breeding. The keywords searched for were, but not limited to, meat chicken, turkey, domestication, breeding, selection, quantitative genetics, genomics, gut, leg, foot, cardiovascular, robustness, behavior, welfare, sustainability, diversity and societal role of meat. The literature cited in this review is concentrated on those papers that influenced the various factors around the development of commercial poultry breeding, including graphs and tables illustrating the achievements and trends. As the information required for a comprehensive overview is only to a limited extent available from electronic literature databases, the information search has also included a wide range of additional sources from, amongst others, physical libraries, government reports, statistical data from government websites, conference proceedings, theses, research reports, websites from audit bodies, professional associations and personal communications. The review of developed technologies has been concentrated on the research that has been implemented in the practice of commercial meat poultry breeding. In the areas of genomics and gut health, recent developments with a view to implementation are also included. Section 1 will cover the history of poultry breeding. Section 2 will detail the development of breeding goals over time from mainly production to including robustness, health, welfare and reproductive characteristics. Section 3 will outline the development of key traits and the management of trait antagonisms, and Section 4 will define balanced breeding for welfare and sustainability.

## 1. History of Commercial Poultry Breeding

Poultry is an affordable meat in most regions of the world. The combined efforts of the poultry sector, coupled with technological and management improvements in a wide range of areas (e.g., poultry health, welfare, nutrition and housing), and particularly poultry breeding advances, have created a world where poultry is now an accessible meat for most of the global population [1,2]. Poultry meat is expected to account for 41% of the protein consumed from all meat sources in 2032, followed by pig, bovine and ovine meat. Poultry production is generally considered more efficient and less resource-intensive, making it a more environmentally sustainable choice of meat [3].

### 1.1. Domestication

The first steps in poultry farming and selection were made when poultry species were domesticated from their wild ancestors thousands of years ago. Chickens originate from the Red Jungle Fowl (*Gallus gallus*), and turkeys from the American wild turkey (*Meleagris gallopavo)*. Domestication occurred at least twice in different regions: the Indus Valley in the Harappan culture 2500 years B.C.E., and at Neolithic sites in Northern China, where there are reports of much older chicken remains (<6000 B.C.E.) [4,5,6,7]. Wang et al. suggest that domestic chickens initially derived from the RJF subspecies Gallus gallus spadiceus in Asia, translocated across Southeast and South Asia, and interbred with other jungle fowl species [8]. The genomes of modern chicken stocks used for meat production confirm the considerable role of heavy Asian breeds in modern broilers [9]. In turkeys, at least two domestication routes from the American wild turkey 800–100 B.C. have also been identified by mitochondrial DNA analyses [10,11].

The process of domestication involved a number of genetic changes: amongst others, selection for reproduction in captivity, for tameness [12] and for the ability to live in large, dynamic groups [13]. Over the last millennia, local varieties were established, with purposes ranging from fighting skills to egg laying or meat production. The modern broiler originates from Cornish, Leghorn, New Hampshire/Rhode Island Red and Plymouth Rock poultry breeds, which were developed in the 19th century in Europe and North America [14,15]. The effects of selection on commercial poultry genetic diversity have been a focus of debate and concern. Muir confirmed via comparative DNA analysis that the decrease in genetic diversity in chickens happened centuries ago, mainly as a result of breed formation, which inevitably results in some inbreeding. Commercial chicken breeding has caused less than one-eighth of diversity reduction [16,17]. Commercial breeding programs maintain effective population sizes to achieve inbreeding rates below 0.01 per generation, e.g., [18].

### 1.2. Development of Science and Commercial Breeding Structures

In the 20th century, major advances in quantitative genetics and its application to livestock breeding stimulated the organizational development and scientific advancements of poultry breeding.

#### 1.2.1. Development of Quantitative Genetics

In 1900, De Vries, Correns and Van Tschermak [19] demonstrated how to link phenotypes with independent inheritance of genes at reproduction. Bateson’s group (1900–1904) [20] showed how morphological traits such as shank color and comb form in chickens were heritable traits and that the inheritance of some traits could depend on gender. In 1908, they discovered the sex linkage of certain genes, which became an important tool later on in the development of the poultry sector [14]. Bateson and Saunders (1902) suggested that traits like body weight are controlled by a large number of genes [2,20]. Fisher (1918) [21] described how the observed continuous variation of traits was influenced by environmental factors and inherited genetic factors. Hardy (1908) [22] and Weinberg (1908) [23] worked out how these principles could be independently applied to large populations. Theories on how to measure and breed for characters such as body weight, hatchability, behavior, disease resistance and ‘linked characters’ emerged in the 1930s [14]. From the 1920s, quantitative genetics was taught at agricultural universities. Since the end of the 1930s, breeding companies started hiring geneticists [2,15].

#### 1.2.2. Testing Stations

At the start of the 20th century, agricultural institutes and research stations started testing birds under standard conditions to separate genetic and environmental factors. For instance, from 1904 to 1911, Punnett and Bailey started body weight inheritance experiments [14,24]. In the 1930s, the United States (US) saw a development of performance recording work on the breeders’ own premises and description of various lines leading to the US private breeders’ Record of Performance Federation work, which was state-supervised. The United Kingdom (UK) worked on poultry improvement at the Northern Breeding Station, the Lancashire Breeding Scheme, the Accredited Poultry Breeding Scheme, and the Approved Cockerel Breeding Scheme. Breeding birds were identified via wingbands (males) and copper legbands (females). Canada registered poultry in the Canadian National Poultry Record Association, an organization of private breeders with a governmental secretariat, performing progeny testing and cooperating with federal and provincial Approved Flock Associations. In addition, other countries like the Netherlands had similar nationally organized breeding improvement structures [14]. These structures resembled the progeny testing programs of dairy cattle and pigs at national and private levels, contrary to the situation in species like sheep and goats where organized structures with data recording and testing of progeny were less or not developed [25,26].

#### 1.2.3. Crossbreeding

Breeding was initially practiced as mass selection, which is selecting individual birds from a mixed population based on their phenotype for traits with sufficient heritability (*h*^2^) like yield [2]. Individual animals were tested for live body weight in early life, and then the best males and females would become the parents of the next generation. The early-life measurement had the advantage that the generation interval could be short, allowing faster genetic progress if compared to egg layers, where the final number of eggs is known at the end of the production life.

With production and reproduction traits being correlated negatively, individual male and female lines were formed, first in layers, e.g., [27], and then also in meat birds. In the 1950s, the testing of crosses for growth rate and meatiness started [2]. Crossing of diverted male and female lines gave an added bonus: extra ‘vitality’ in the crossed generation, a phenomenon recognized by and implemented in plant and animal breeding. This so-called heterosis effect (or hybrid vigor) [14] is highest in the first generation (F1) and decreases in the second (F2) and further generations [2,24].

**Figure 1:**
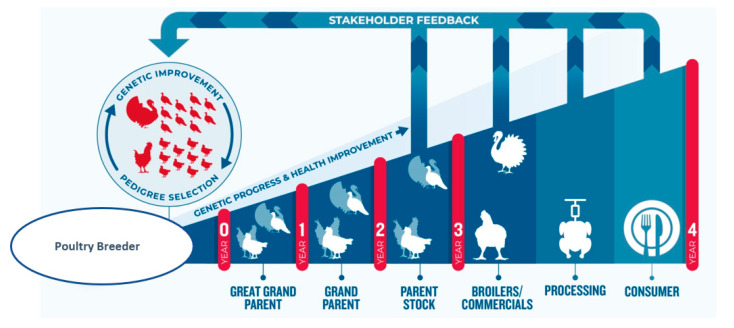
Breeding structure/pyramid. Selection of pedigree lines, multiplication pyramid and feedback mechanism of meat poultry breeding program. Next to the pedigree populations that contribute directly to the crossbreeds on the market, there are control lines that have not been developed since the 1970s or 1990s. The development and research lines are useful as reference populations or may contribute to future crossbreeds. Adapted from [28].

**Figure 2:**
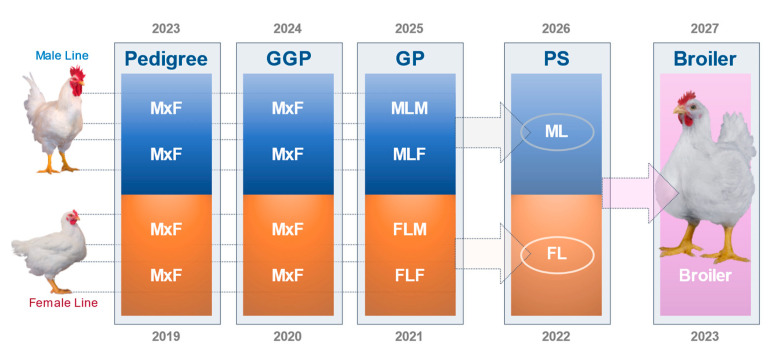
Multiplication: From pure lines to commercial crossbreds. Multiplication from pure lines to commercial crossbreeds in broiler breeding. A similar structure is used in turkey breeding. M—Male; F—Female; MLM—Male line male; MLF—Male line female; FLM—Female line male; FLF—Female line female; ML—Male line; FL—Female line; GGP—Great Grandparent; GP—Grandparent; PS—Parent Stock.

Crossbreeding requires structure and investment as the contributing flocks to the cross and the resulting hybrid must be maintained. Jull [14]: “The poultry breeder would have to breed two distinct flocks and provide room for the hybrid (crossbred) progeny”. Nowadays, a range of pedigree lines are selected for a broad range of traits and offspring are multiplied and crossed over several generations. From pedigree selection through to the commercial generation, grown by farmers takes around 4 years. Figure 1 shows a typical poultry supply chain structure, the so-called breeding pyramid. The genetic improvement takes place in the pure lines from which it then disseminates to the rest of the industry through a series of multiplying generations. Crossbreeding typically starts at the grandparent stock level, where pure lines are combined into a variety of crossbreeds to meet the needs of different markets (Figure 2). The breeding program sits at the start of the supply chain and receives continuous feedback from a range of sources, allowing the setting up and fine-tuning of relevant breeding goals.

#### 1.2.4. Pedigree Breeding Structure

The next step in the evolution of breeding programs involved the inclusion of information from relatives, e.g., full sibs (FS) and half sibs (HS), in the breeding value estimation of individual birds [29]. This step required the building up of pedigree information of populations. Jull [14] explained the importance and necessity of pedigree breeding to poultry breeders, the development of a breeding program based on progeny testing being the surest way of making progress. He indicated that “only a relatively few poultrymen are properly qualified to undertake pedigree breeding work”. This describes remarkably well the essence of a modern breeding program: large amounts of data are recorded on each of many birds; these carefully recorded measures are combined with the birds’ pedigree to assess each individual’s merit.

The typical hierarchical structure of poultry breeding programs, with a number of females, mated to a single male, was first exploited statistically by Osborne (1957) [29]. This led to significant increases in the selection accuracy for a host of characteristics. However, the “Osborne Index”, being a univariate approach, is not optimal for sequential selection structures where a first selection based on a large group is followed by one or more successive selection steps in the remaining smaller group of preselected animals. Therefore, a new prediction method was needed.

Henderson (1975) [30] explored breeding from a statistical point of view in even more depth, estimating genetic parameters and combining phenotypic performance and pedigree relationship to maximize the genetic gain in a breeding program. The so-called Best Linear Unbiased Prediction method (BLUP) enabled estimating the breeding value of potential selection candidates based on progeny performance to select superior genotypes and breed superior families. BLUP is well suited for multivariate, sequential selection settings as generally applied in poultry. This required considerable computing power, which became available in the early 1980s when the first university curricula started exploring BLUP applications for the major farm animal species like cattle, pigs and poultry. The family information built up over time is extensive for each of the breeding programs; for example, the Aviagen broiler pedigree goes back to the late 1970s.

#### 1.2.5. Development of Genomic Selection

From the 1970s, estimations of the genetic makeup of living organisms using genomics information started to become available. Bacterial and human genome sequences were published in 1976 [31] and 2001 [32], respectively; the chicken genome in 2004 [33], and the first part of the turkey genome map followed in 2012 [34]. The use of genomics information for selection purposes required the development of statistical frameworks that allowed estimating the variance accounted for by genomics information and the prediction of marker effects (either individually or across the whole genome). Following the seminal papers on genomic selection [35,36] enabling the use of genomics information in a quantitative genetics framework, genomic selection is now a tool available across livestock populations, including poultry. Further detail on the development and implementation of genomics into meat poultry breeding programs is described in Section 3.3.1.

Integrating phenotypic and genomic information from pedigreed animals into smooth-running breeding operations in a timely and highly accurate fashion requires high throughput data collection and processing. While a range of tools and methods are available, the development of statistical tools to enable the management of the growing complexity of data and their relationships is a continuing process.

#### 1.2.6. From Specialist Lines Breeders to Broad Spectrum Breeders

Early in the development of commercial poultry, breeding companies focused on one sector of the market and specialized in developing one or a few crossbred population(s).

There was a relatively large number of “one product” companies—and that specialization was initially a strength, and the success or failure of the company was very much linked to that specific crossbreed. With 10–20 companies and 10–20 corresponding crossbred offerings, only a few would be suitable for the market needs at any point in time. With this model, companies were unable to adapt easily to market changes, resulting in large shifts in breed dominance over time and across world regions [15]. In addition, from the 1980s, larger investments in research and development (R&D) and breeding operations were needed to remain competitive. This resulted in the amalgamations of many specialized single-line breeders to a lesser number of breeders with multiple lines and sometimes multiple species or types [15].

## 2. Evolution in Breeding Goals

Genetic selection is the identification of the most appropriate birds to become the parents of the next generation. The breeding goal determines what “most appropriate” means in practical terms [37]. Breeding goals have expanded vastly in the last four decades from production only to balancing production, reproduction, health, welfare and environmental impact, simultaneously improving these aspects [17,37,38].

### 2.1. Breeding Goal

The breeding goal defines the set of traits aimed for improvement in a specific animal population. Modern poultry breeding programs include broad gene pools consisting of tens of genetic lines with specific breeding goals depending on their role in the commercial crossbreed.

The breeding goal of a genetic line lays out precisely the directional improvement desired in each trait. For each trait in the breeding goal, each selection candidate gets an estimated breeding value (EBV) combining its own phenotypic information plus information from contemporary relatives and ancestors. Selection accuracy is a function of all the information available at the time of selection and the genetic basis of the trait, and it is a central factor for predicting response to selection and genetic trends that ultimately determine the success (or failure) of a breeding company.

Modern crossbred populations are typically derived from the crossing of three to four genetic pure lines (Figure 2). The combination of these lines and the performance of the different combinations in the field determine which commercial crossbreeds are available in the market. The balance of selection traits in each line differs depending on its role in the final commercial cross; this balance can range from focusing on growth, yield and biological efficiency to reproduction. For instance, in a line contributing to a slow-growing crossbreed, there will be a low or no emphasis on ‘growth rate’. Today, animal health and welfare traits are included in the balance of selection traits across the whole portfolio of pure lines, contributing to a commercial cross [17,28,37,39].

The setting of breeding goals requires a long-term view to ensure the sustainability of breeding improvements and the ability to adapt to changes in market requirements and incorporate feedback through the supply chain mentioned in Figure 1.

### 2.2. What Determines a Breeding Goal

External factors will influence how breeding goals develop, which will likely vary depending on the different market segments and geographies. For example, the emphasis on different traits in the breeding goal will change depending on the live weight or yield requirements (e.g., whole yield or parts) of the final crossbred population or whether there is more or less emphasis on welfare requirements. Global poultry meat production and consumption patterns (e.g., as a function of Gross Domestic Product (GDP) as illustrated in [3]) will indeed influence the architecture of breeding goals and the relative emphasis on different traits. Therefore, breeding companies need to carefully anticipate the direction of global developments and stakeholder requirements to satisfy future requirements, as the requirements of the market and society are intrinsic parts of the feedback into breeding programs (Figure 1). The many possible future scenarios need to be taken into account when making optimal crosses that serve evolving market needs. Hiemstra and Ten Napel [37] investigated three potential policy scenarios for the European Union (creating a better match between breeds/lines and the environment, maintenance of genetic diversity, monitoring of broiler welfare in the production chain) and a business-as-usual scenario. They found that legislative oversight of breeding goals would likely hamper the development process toward better welfare or diversity and/or move breeding work to other regions, which would decrease the influence on directional population change. It would also hamper the development of R&D investment. However, a mandatory scheme to collect and publish data in commercial slaughterhouses or production farms would facilitate a market-driven, outcome-based approach to improving broiler welfare [37].

Neeteson-van Nieuwenhoven et al. [17] discussed the role of monogastric breeding in food security and highlighted that with the increased demand for animal products and decreasing availability of resources such as land and water, livestock production needs to increase productivity and reduce environmental impact. This is in line with projections by OECD-FAO [3], which forecasted an increase in global meat production of 15% by 2032 but with only a 7.6% increase in greenhouse gas emissions (GHG) due to the increased share of poultry meat, which is predicted to be 41% of the total meat production by 2032. Biological efficiency in terms of the meat-to-feed ratio will continue to be a key driver in current and future breeding goals. In addition, Neeteson-van Nieuwenhoven et al. [17] also concluded that for that reason, “feed availability and environmental load form true constraints to livestock production (e.g., more production means more nitrogen excretion), but not to animal breeding: improvement of the traits related to these issues goes hand in hand with improvement of productivity (e.g., greater animal productivity means decreased nitrogen excretion)”.

### 2.3. Expansion of Breeding Goals

**Figure 3:**
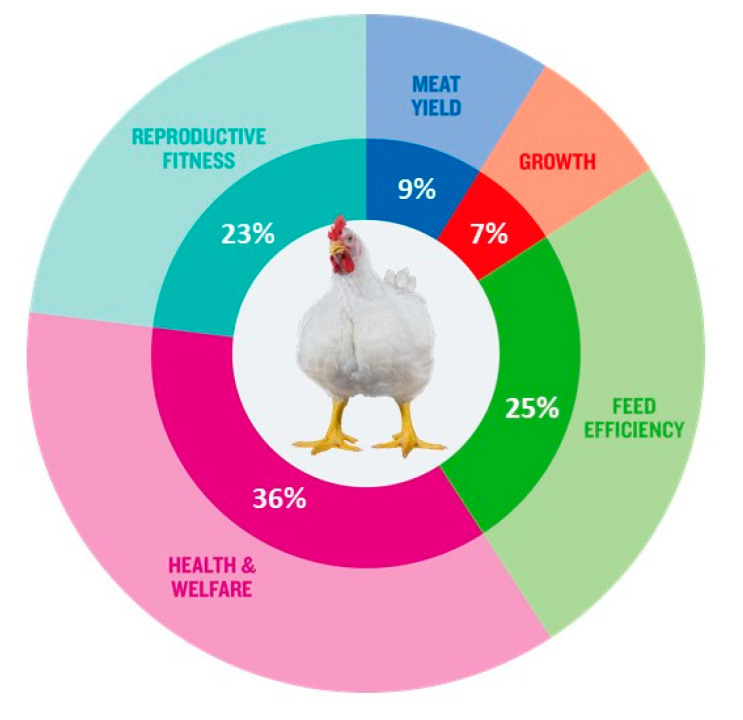
Example breeding goal Ross 308.

Neeteson-van Nieuwenhoven et al. [17] highlighted that breeding goals should be broadened in a balanced way, focusing on productivity and efficiency but subject to constraints due to agricultural resources, environmental load, and animal welfare as well as to possible restrictions due to genotype by environmental interactions, antagonisms between traits and potential selection limits. The availability of more powerful computing power enabled the handling of vast amounts of data, leading to high throughput phenotyping. Coupled with strong investment in R&D, this has allowed breeding goals to expand from focusing on a single or reduced number of traits in the 1950s to multiple trait goals [15,17,40]. Modern breeding programs consist of tens of traits across a very wide range of bird performance and attributes related to production, reproduction, robustness, health, product quality and environmental adaptability. In [17], the authors illustrated that “commercial poultry and pig breeding goals have broadened widely since the 1970s, typically including 30 to 40 traits now. More traits are to follow because of continuous trait development, increased data recording efforts, and increasingly powerful statistical methods”, following a survey amongst animal breeding experts worldwide on the relative importance of elements of pig and poultry breeding goals over time. This survey indicated that health and livability traits featured in breeding goals are as important as animal welfare and productivity traits, thus not representing a constraint to an increase of productivity: “Many of the adaptability traits will form a novel element in breeding goals”. Hiemstra and Ten Napel [37] reported in 2013 that the relative weighing of welfare traits in the breeding goal varied between 18–33% across broiler breeding companies. Figure 3 shows that since then, the impact on health and welfare traits regarding breeding goals has increased in a conventional (‘fast growing’) broiler breed, the Ross 308.

The process of breeding goal expansion is expected to continue [41]. Continuous trait development, increased data recording and automation efforts, and increasingly powerful statistical methods will further drive the broadening of breeding goals [17]. These novel opportunities enable the improvement of health and welfare, environmental impact and productivity at the same time.

## 3. Genetics Basis of Key Traits and the Management of Antagonisms

In a multi-trait breeding goal, there is a wide range of traits with different genetic variability (heritability, *h*^2^) and genetic correlations between them, leading to neutral, positive or antagonistic relationships.

### 3.1. Traits

Traits recorded in a breeding program are either breeding goal traits or predictor traits that have correlations with breeding goal traits. EBVs are predicted for each trait after estimating *h*^2^s and genetic correlations using animal models in a mixed-model equation framework, utilizing all the information available, including each bird’s own performance and family phenotypic information.

Phenotypes range from traits that require minimum recording cost, like body weight at different ages, livability, leg health and physical examinations that only require operator training, to more sophisticated and expensive traits to record, like reproduction (egg production, fertility and hatchability), feed efficiency, meat yield and meat quality and indeed metabolic measurements like cardiovascular and gut function and skeletal integrity. Over the years, there have been several examples of highly sophisticated technological developments to record high-throughput phenotypes for breeding goal traits: i. X-ray technology for the detection of the clinical and sub-clinical incidence of Tibial Dyschondroplasia (TD); ii. Transponder technology uses Radio Frequency Identification (RFID) to record feed and water intake in large groups of birds and understand the genetic basis of feed and water behavior, e.g., [42,43]; iii. Two-dimensional ultrasound to predict meat yield in the live bird; iv. Pulse Oximeter to record oxygen saturation levels in blood (SaO_2_) and heart beat rate; v. Computed Tomography (CT) to predict yield, body composition and assess skeletal soundness in live birds.

These traits require significant technological developments and R&D investment and often bring technology from other disciplines like medical science. An example of a new frontier in trait recording is predicting gut function in live birds and the impact of the gut microbiome on biological performance. The availability of blood biomarkers, whole bacteria sequencing, and a framework to fit this novel information in genetic evaluations will enable further expansion of breeding goals because they underlie complex biological processes, for example, behavior, immune response and gut function.

As mentioned before, traits that affect the level of welfare have a significant weight in the breeding goal, and very important efforts have been made, resulting in improvements in a growing number of characteristics covering health and welfare since the 1970s.

**Figure 4:**
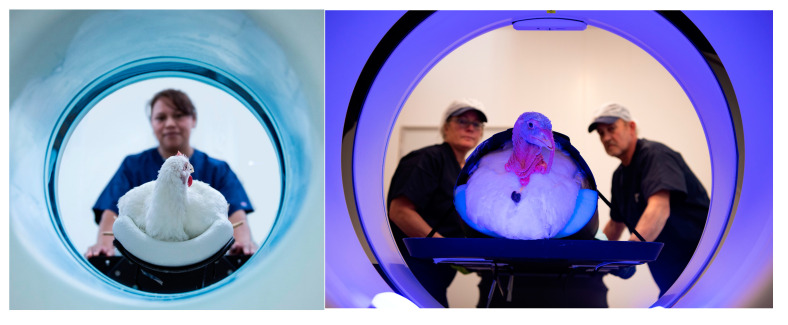
CT scanning of broilers (**Left**) and turkeys (**Right**).

The recent introduction of CT and 3D imaging technology in broiler and turkey breeding provides unprecedented opportunities to unlock information about the body systems of the bird and generate novel phenotypes from the live bird. As well as allowing for accurate predictions of meat yield, CT allows live bird assessments of skeletal integrity (Figure 4) and internal organs, leading to a full-body atlas.

### 3.2. Development of Key Traits

Given the recent focus on health, welfare, sustainability and quality, we will focus in more detail on the development and direction of traits related to these three key areas of breeding goals.

#### 3.2.1. Traits Covering Health and Welfare

Leg health

**Figure 5:**
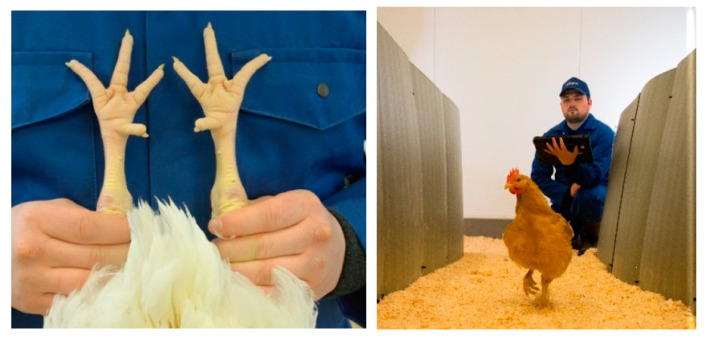
Leg and foot health (**Left**) and gait (**Right**) assessment in broiler selection candidates.

**Figure 6:**
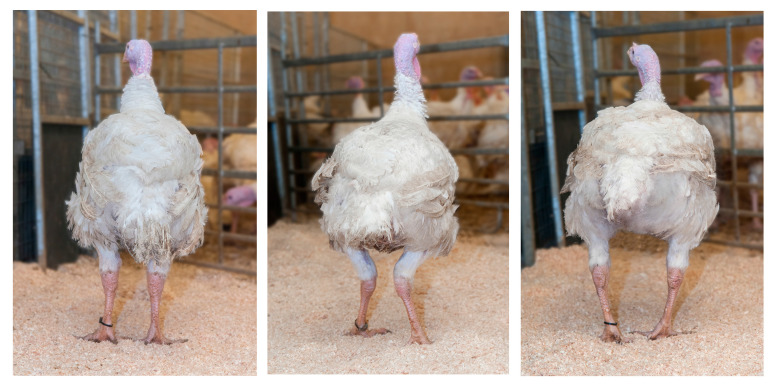
Images of gait scoring in turkeys. (**Left**): Healthy legs compared to (**Middle**): valgus, and (**Right**): varus deformities.

Leg health has been a key feature in Aviagen’s breeding programs since the 1970s. Every selection candidate goes through a very detailed examination of leg health to detect valgus/varus defects and long bone deformities. This began with the removal of birds with any clinical leg defects. Nowadays, a walking assessment (gait scoring) is also part of leg health assessment in broilers and turkeys. Along with leg defects, each bird is also screened for contact dermatitis, footpad dermatitis (FPD), hock skin lesions and toe defects. Aviagen adheres to a zero leg-defect tolerance policy, meaning that any bird displaying any type of leg defect is not considered for selection or used for breeding and, therefore, will not contribute to the next generation. This policy has been a driving factor in reducing the genetic propensity of the development of leg defects in chicken and turkey breeding populations, as demonstrated by Kapell et al. in 2012 [44] in terms of broilers and Kapell et al. in 2017 [45], in terms of turkeys. The addition of family-based selection has also made it possible to exclude defect-free individuals from high-defect incidence families. Figure 5 shows the leg and foot health and gait assessments currently undertaken in broilers. Figure 6 shows the gait assessment in turkeys.

**Figure 7:**
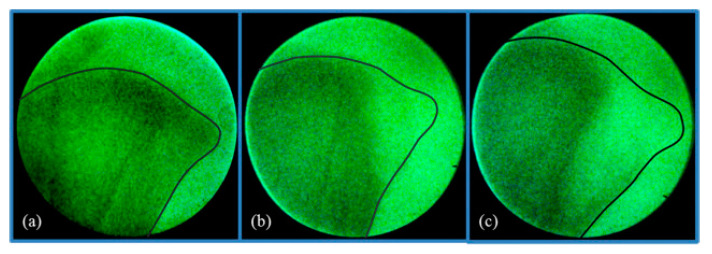
Lixiscope X-ray images showing Tibial Dyschondroplasia assessment in turkeys. (**a**) No lesions, (**b**) moderate lesions, and (**c**) severe lesions. Source: [35].

The scope of leg health assessments has expanded over the years to include technology such as the pioneering use of a hand-held X-ray device (Lixiscope) for the detection of clinical and sub-clinical TD (reduced mineralization at the proximal end of the tibia, Figure 7). This work began initially in broilers in 1989. New-generation Lixiscopes, introduced in the late 2000s, improved the accuracy of detection and also made it possible to apply this technology to turkeys where, alongside gait and defect assessment, it continues to be used today.

**Figure 8:**
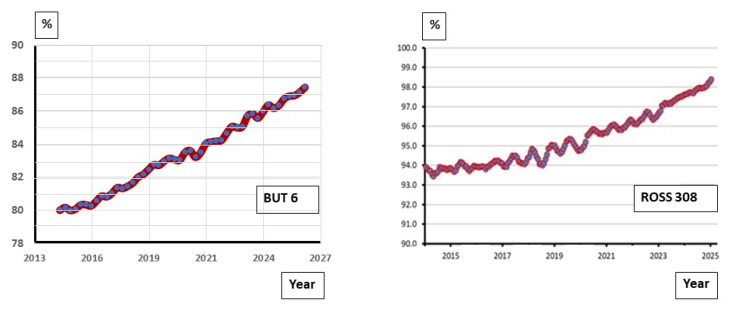
BUT 6 (**Left**) and Ross 308 (**Right**) genetic trend for selected leg health traits. *X*-axis: customer year. Y-axis: Leg Defect % Free. For BUT 6 and Ross 308, the genetic trends depict the improvement in % leg-defect-free birds, including information from clinical and subclinical leg health assessments and gait evaluations.

The inclusion of a range of leg health traits in the breeding goal has generated genetic trends showing improved leg health across a number of traits (Figure 8).

**Figure 9:**
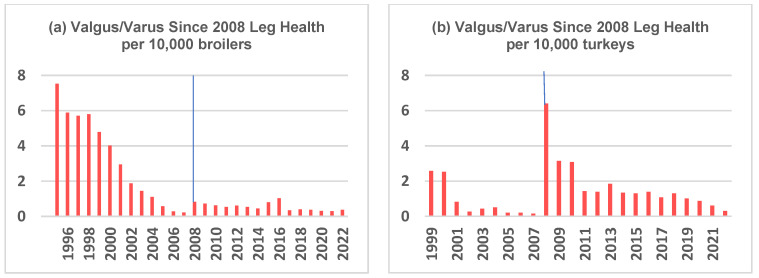
Leg health (until 2007 valgus/varus) related condemnation rates in broilers and turkeys per 10,000. (**a**) Chickens 1995–2022; (**b**) Turkeys 1999–2022 [46]. The vertical blue lines mark the change from valgus/varus to leg health in 2008.

Improvements in leg and toe integrity are confirmed by independent data from Agriculture and Agri-Food Canada (AAFC) [46] (Figure 9), peer-reviewed literature (e.g., leg strength and toes in Kapell et al. [47], the FAWC (2012) report [40], and the EC broiler breeding study [37]). Figure 9 shows the Canadian improvements in leg and toe integrity from 1995 (chickens)/1999 (turkeys) to 2022. In 2008, the criteria changed from ‘valgus/varus’ to ‘leg issues’, which explains the temporary increase in 2008.

In recent decades, welfare research on contact dermatitis shifted gradually from hockburn (measured and selected against since the 1970s) to FPD, a commonly used welfare indicator. Genetic selection to improve turkey and broiler FPD began in 2008 by scoring footpads on every pedigree individual and selecting individuals that show a low genetic predisposition to develop FPD. Kapell et al. [47] explained how genetic selection in contrasting environments is an effective tool to achieve this.

**Figure 10:**
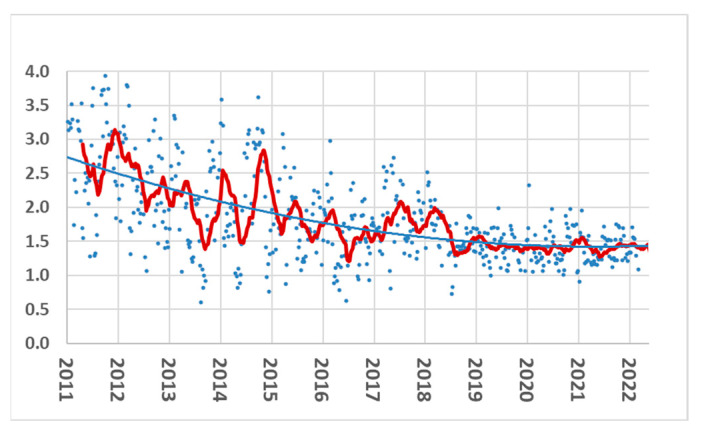
Trend graph showing FPD (average score) in BUT 6 Pedigree birds in the pedigree environment. Scoring: 0 = clear, no FPD; 1 = less than 25% of the pad; 2 = less than 50% of the pad; 3 = greater than 50% of the pad; 4 = pad and toepads affected. FPD—footpad dermatitis.

Wet litter is also a key contributor to the incidence of FPD in turkeys [48,49,50,51]. In 2011, Aviagen Turkeys implemented individual water intake measurements using technology similar to its feed stations to identify birds with excessive water consumption, which has been shown to contribute significantly to litter moisture. The combination of the targeted exclusion of individuals creating wet litter as well as those with a higher tendency to develop FPD is an effective genetic means of improving the bird’s footpad health for the future, as is illustrated for turkeys in Figure 10.

**Figure 11:**
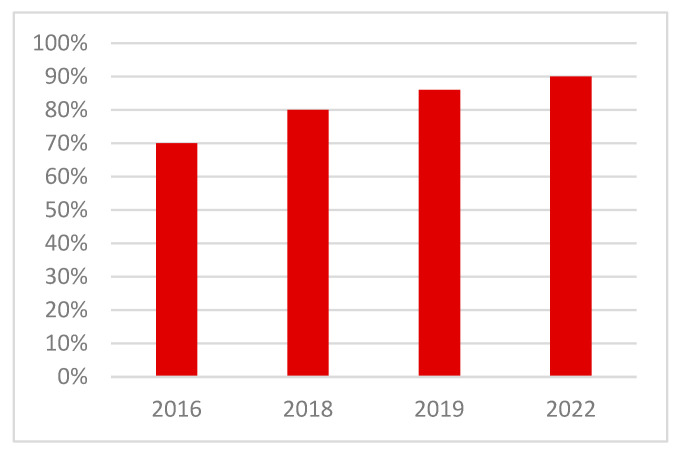
% Birds with gait scores of Ross 308 broilers (Bristol scores 0–3). Fixed weights at 2.3 kg, RSPCA method [52]. Aviagen trials farm. During 2020 and 2021, no measurements could be made due to COVID-19-related travel restrictions.

Furthermore, in the chicken breeding program, improvements in gait scores are clear (Figure 11). Ross 308 broilers showed a steady gait improvement from 2016 to 2022.

Heart and lung strength/fitness

**Figure 12:**
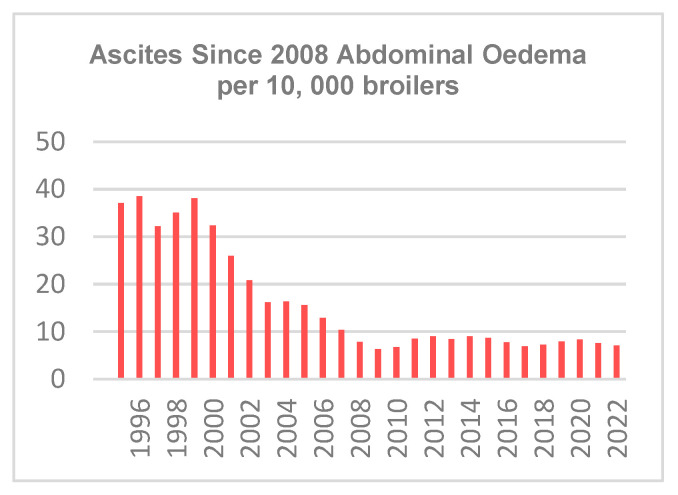
Ascites-related (as from 2008 abdominal edema) condemnation rates in broilers per 10,000. 1995–2022. Source: [46].

Since 1991, Aviagen has been evaluating cardiovascular health using pulse oximetry to measure the level of oxygen saturation in the blood of pedigree birds. This is an important indicator of the susceptibility of an individual to develop ascites (accumulation of non-inflammatory fluid in the abdominal captivity) and sudden death syndrome. Ascites levels, as measured by AAFC, have decreased substantially over time as a result of the joint effect of breeding and improved management (Figure 12). As ascites prevalence in turkeys has always been low, the Canadian turkey trend is not shown.

Livability

**Figure 13:**
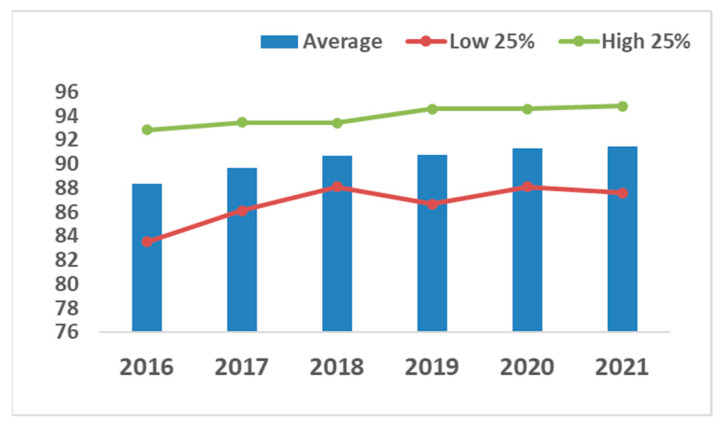
Field livability of BUT 6 commercial males from a European turkey producer. Average livability and the average of the highest and lowest 25% of flocks for each year. Results from approximately 170 flocks each year.

Livability is a key trait for both the welfare and sustainability of poultry production. Improvements in livability are targeted through recording mortality. Mortality is recorded at all stages of the production cycle and in high- and low-hygiene environments as part of robustness and environmental adaptability selection [53]. Livability is also indirectly improved through the selection of leg health and cardiovascular function. The genetic improvement of field livability is achieved using a broad breeding goal combining mortality information with production, yield and biological efficiency. Figure 13 shows improvements in field livability over time for commercial BUT 6 male turkeys in Europe. In chickens, the authors of [11,37] reported livability increases within broiler lines and crossbred populations ranging 0.2–1.0% per year.

Behavior: feeding and drinking behavior, other behaviors

**Figure 14:**
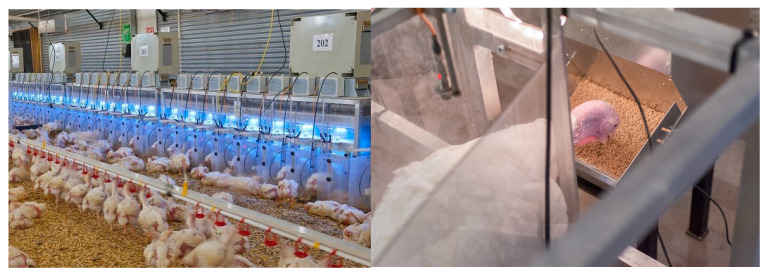
Feeding station: broilers (**Left**), turkeys (**Right**).

With the continuous globalization of poultry meat production and concerns related to limited natural resources, the genetic improvement of biological efficiency will continue to be a central focus in broiler and turkey breeding goals. The use of electronic feeders and drinkers, so-called feed and water stations (Figure 14), combined with RFID transponders, has allowed the feeding of individual broilers and turkeys to be recorded. Drinking patterns have enabled an understanding of feeding behavior structure across various species and the estimation of the genetic basis for feeding and water efficiency [42,43].

Howie et al. (2010) [54] found that broilers, turkeys and ducks share the same structure of short-term feeding behavior, which is regulated by levels of satiety. This was also observed when comparing broilers, turkeys and ducks to cattle, pigs, dolphins and rats [55]. These results indicate that selection for feed efficiency has not had a correlated response to feeding behavior traits.

**Figure 15:**
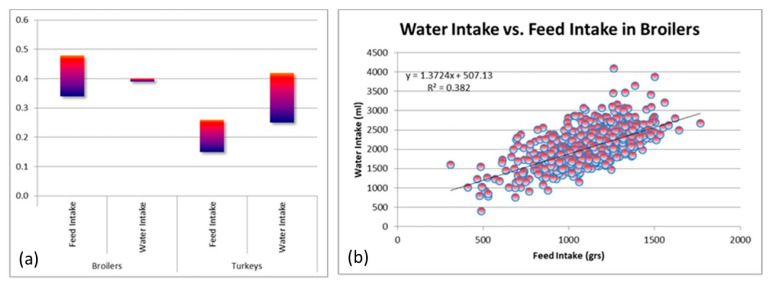
(**a**) Heritabilities for Feed Intake (FI) and Water Intake (WI) for broiler and turkey lines from the Aviagen breeding programs; (**b**) Relationship between WI and FI in broilers (measured from 14 to 35 days). Source: [53].

Figure 15a shows the *h*^2^*s* of Feed Intake (FI) and Water Intake (WI) for a range of broiler and turkey lines in the Aviagen breeding programs. The *h*^2^ for FI in broilers ranges from 0.34 to 0.48; in turkeys from 0.15 to 0.26 [53]. The lower *h*^2^ range in turkeys could be related to the highly contrasting testing age ranges and length of testing periods for broilers up to 47 days and turkeys up to 18 weeks. For WI, the *h*^2^ in broilers and turkeys ranges between 0.27 and 0.47, with a much narrower range in broilers. These ranges indicate a wide range of genetic variation available in modern broilers and turkeys for the improvement of biological efficiency. Figure 15b shows the phenotypic relationship between WI and FI for broilers between 32 and 42 days of age. It is very interesting to note the wide range of WI observed for a given level of FI. For example, for FI around 1 kg, there are birds drinking between 1 and 3 L of water. A key component of the selection strategy for overall biological efficiency consists of selecting out birds that consume excessive levels of water at the same level of FI. Birds with excessive levels of WI are also likely to have poorer gut function and health, make the litter wetter, and contribute to a higher incidence of contact dermatitis and FPD in the flock. Thus, these birds have lower rates of welfare and will also have a more negative impact on the environment.

Interestingly, Howie et al. (2011) [42] and Rusakova et al. [43] found low correlations between feeding and drinking behavior traits and performance traits. Given this low correlation, directional selection for biological efficiency is independent of the expression of feed and water-drinking behavior. Thus, broilers and turkeys can express a wide range of feed and drinking behavior strategies to achieve a given level of biological efficiency, which is a critical component of their adaptability to a wide range of environments and production systems.

The management of feed intake of broiler breeders during puberty is often raised as a welfare concern in poultry production. There is a negative correlation between broiler and breeder traits, sometimes called the broiler–breeder paradox [56]. Broilers are selected for better FCR and growth potential at a young age. Breeders are offered adapted feeding programs so that they grow less meat and are more reproductive, allowing them to develop the necessary abdominal fat without becoming too muscular. The genetic correlations between early and later growth, and similarly between early and late appetite, are very high [57]. It is possible to manage the chicken growth curve by selection [58], but at the cost of very large sacrifices in broiler gain because of the high genetic correlation with late gain. Increasing mature weight and the animal’s option to achieve that is not desirable when considering the reproductive capability of these animals. Dawkins and Layton [59] argue that by changing selection goals, sampling other populations and incorporating appropriate quantitative trait loci (QTLs) from non-pedigree populations, the antagonism could be solved, but the feasibility of this is not clear. Cross-bred combinations of fast-growing male lines with slower-growing female lines have indeed addressed an important part of the issue, with the slower-growing females being fed closer to ad libitum levels in both rear and lay without elevated levels of mortality or multiple-ovulation. Currently, management changes, e.g., feed intake control during puberty, are also a practical and effective approach to managing the antagonism. The European Food Safety Authority (EFSA) (2009) [60] (p. 23) concluded the paragraph on breeder feed intake management as follows: “However, research in this area is very limited and more research is necessary to draw firm conclusions about feeding programs in relation to bird welfare”. Since then, increased research efforts on alternative feeding systems and their impact on feeding amounts, behavior and stress indicators have shown promising results of feed intake management in the areas of, amongst others, feed dilution and feeding twice per day on the health and welfare of breeders in rear and lay, e.g., [61,62,63,64,65,66,67,68,69,70]. Research and development will fine-tune broiler breeder feeding management further toward implementation.

#### 3.2.2. Environmental Impact Traits

Environmental sustainability has long been a core focus in modern poultry breeding. While increasing flock outputs through improvements in traits associated with live weight, livability, egg production and meat yield play a role, the feed conversion rate, defined as the ratio between feed intake over weight gain (FCR), is the single most important trait for reducing the environmental impact of poultry production [71].

Feed conversion rate

**Figure 16:**
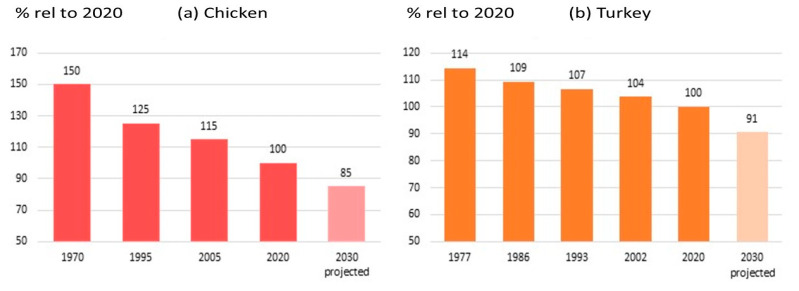
Impact of genetic improvement on CO_2_ emissions (Global Warming Potential), relative (rel) to 2020, to a fixed weight (BUT6 20 kg; Ross 308 2.5 kg) and including a future projection to 2030. FCR—Feed Conversion Rate. Feed Conversion Rate (FCR) is the major contributor to reduction in Global Warming Potential. Sources [27,72].

The improvement of FCR in both broilers and turkeys has greatly reduced the carbon footprint of poultry meat and substantially reduced the amount of environmental pollutants associated with poultry production. Figure 16a shows the relative environmental impact of broiler production (measured as % carbon footprint, kg CO_2_/kg eviscerated weight) over time. Aviagen estimates that using Life Cycle Analysis (LCA) modeling shows that the broiler genetics from 1972 had a 50% higher environmental impact than 2020 genetics and predicts a further 15% reduction in carbon footprint by 2030, which is in line with the estimations made by Jones [71]. Turkey genetics resulted in a 20% lower carbon footprint between 1977 and 2020, with an expected 10% reduction by 2030 because of increased focus on FCR (Figure 16b). As indicated above, this progression of about 1% per year is driven primarily by genetic improvement of FCR.

**Figure 17:**
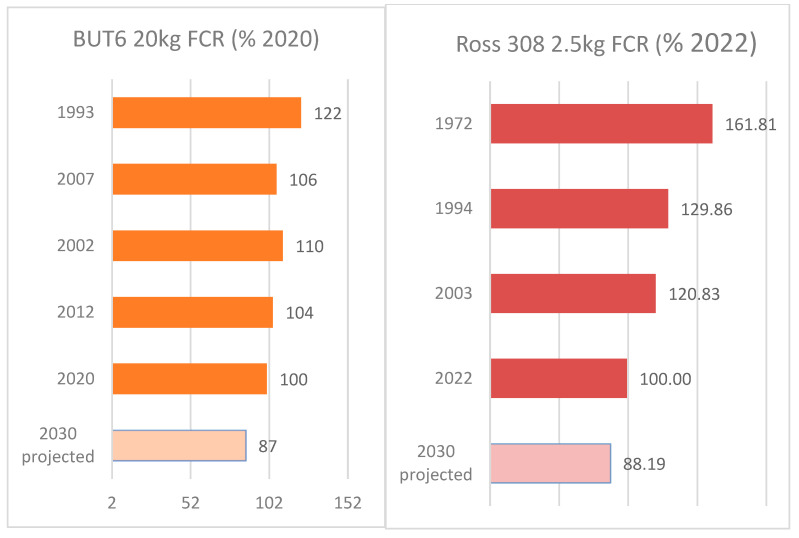
Aviagen published performance objectives for BUT 6 (**Left**) and Ross 308 (**right**) showing FCR performance, relative to 2020 (BUT 6) and 2022 (Ross 308), to a fixed weight (BUT 6 20 kg; Ross 308 2.5 kg) and including a future projection to 2030. FCR—Feed Conversion Rate. Source: [73,74,75,76,77,78,79,80].

For decades, intensive selection for improved FCR has resulted in a highly feed-efficient animal far more environmentally sustainable than many alternative meat sources. This can be seen in FCR improvements outlined in the performance objectives published for the BUT 6 and Ross 308 (Figure 17). Performance objectives are customer guides that list the expected performance in a range of traits based on the current genetic potential of the birds. Since 1993, BUT 6 FCR has been reduced by 0.51 in turkeys, representing a 10.2 kg (18%) feed intake reduction for a 20 kg bird. In Ross 308 broilers, the feed required per kg has reduced by 0.89 kg, representing a 2.2 kg reduction in feed intake since 1972 for a 2.5 kg bird. These long-term improvements represent about 20 g less feed required per kg live (−0.02 kg/kg) weight in both turkeys and chickens. In addition to the benefits of FCR genetic improvement in reducing environmental impact, it also has a direct impact on the agricultural resource requirements for poultry production. An FCR improvement of −0.02 for a broiler integration processing of 1 M broilers per week with a target processing weight of 2.5 kg represents a yearly savings of around 2600 tons of feed, 4700 tons of water, thus releasing 557 agricultural hectares (maize, wheat and soya combined).

#### 3.2.3. Robustness Traits

Given the global nature of poultry production and the great diversity of geographies and production environments, it is crucial that poultry breeding focuses on selection for robustness and environmental adaptability through the management of the genotype by environment interaction (GxE). A key component of good welfare is the ability of birds to thrive in a variety of environments. Robustness or little dependence on favorable conditions or management is incorporated into the breeding goal by evaluating relatives of the birds under selection in a less favorable environment in terms of feed, health, farming conditions or management. As pedigree birds contribute to future generations, the pedigree facilities are maintained to the highest biosecurity standards (see also Figure 1), also known as High Input (HI). This means that pedigree birds are allowed to express their genetic potential in the absence of challenges usually found in commercial poultry production. To measure the potential of birds when grown under natural health and gut challenges, a parallel farming system can be used where siblings of pedigree birds are grown and assessed in lower hygiene conditions or Low-Input (LI) environments. Pedigree selections are then based on performance measurements from both locations (multi-environment selection), ensuring that only the families that perform well in both types of environments pass their genes on to the next generation. This process started in broilers in 2000 and turkeys in 2010. Over time, this process of ‘multi-environment selection’ has had a dramatic effect on the robustness of various management and immune challenges, ensuring a higher level of health and welfare as experienced by the birds.

One of the mechanisms for improving health and welfare through multi-environment selection is the selection for improved robustness of the digestive system. By selecting for feed efficiency under challenging conditions, the development of healthy, well-formed intestinal walls within the pedigree bird populations is improved. This robust gut, a factor of improved feed efficiency, also protects the bird from infection, as the gut is the area of the bird most exposed to pathogens it may encounter in its environment.

**Figure 18:**
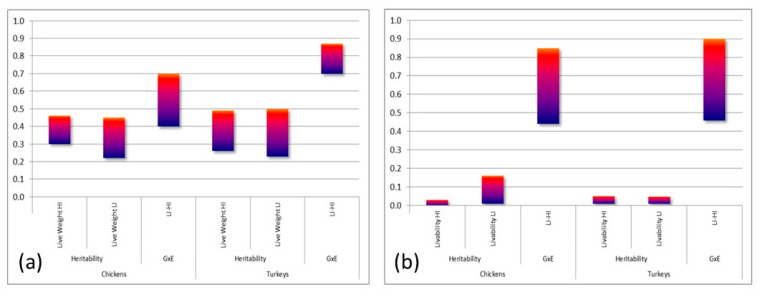
Heritabilities and genetic correlations for live weight (**a**) and livability (**b**) in high-input (HI) and low-input (LI) environments in broiler and turkey lines from the Aviagen breeding programs. GxE—Genetics by Environment Interaction [53].

Figure 18 shows the ranges of *h*^2^ and genetic correlation (GxE) between environments for live weight (LWT) and livability (LIV) for a range of chicken and turkey lines within the Aviagen breeding programs. The *h*^2^ of both LWT in broilers (35 days) and turkeys (18 weeks) ranges between 0.2 to 0.5 in both HI and LI environments, indicating ample opportunities to improve this trait in each environment. The GxE for LWT in broilers (0.4–0.7) has a lower range than in turkeys (0.7–0.88). As expected, the *h*^2^ of LIV is much lower compared to LWT. Broilers have a wider range of *h*^2^ in the LI environment. The GxE range for LIV is similar in both broilers and turkeys, ranging between 0.45 and 0.9. Overall, there is a wide range of genetic variation in each environment, but crucially, to improve environmental adaptability and robustness, the same biological trait should be treated as two different genetic traits in the breeding goal to account for GxE. The improvement of adaptability and environmental robustness arises from identifying genetic lines and families within lines that perform well across environments.

#### 3.2.4. Meat Quality Traits

Selection for breast meat yield has been a core tenet for primary breeders as part of the strategy to obtain year-on-year improvements in bird performance and biological efficiency. As mentioned above, balanced breeding goals are critical to mitigate the potentially undesirable consequences of genetic selection on bird health, and the same is true for carcass and meat quality. The muscle tissues, as with all biological tissues, can demonstrate pathology, which can impact the quality of breast meat and lead to economic losses [81]. Breast myopathies are, therefore, an important concern across the poultry industry. One of the first myopathies reported was deep pectoral myopathy (DPM) in the 1970s and 1980s [82,83,84], and more recently, three novel myopathies have been reported in broiler chickens, which have been termed “wooden breast” (WB), “white striping” (WS) and “spaghetti breast” (SB) [85]. These myopathies are easily detected and distinguishable, giving the opportunity to record them on selection candidates [86]. DPM, WS and stringy spongy (SS) can currently only be detected during bird processing and carcass evaluation, whereas WB can also be detected in the live bird through palpation of the breast meat. All the myopathies are recorded on a categorical severity scale on wingbands, and the data is then used to determine a bird’s genetic propensity for developing the myopathies as part of the selection strategy [81,86].

Recent estimations of the genetic basis have shown that the *h*^2^*s* of the myopathies are low to moderate (0.021–0.06 for DPM, 0.04 for SB, 0.024–0.097 for WB and 0.185–0.338 for WS), indicating that the non-genetic effects are the major influencing factor [81,86]. Understanding the non-genetic factors and implementing strategies to mitigate their impact has significantly reduced myopathy incidence in the field; these strategies have included technical advice on nutrition, bird management, and the handling of birds before, during and after bird processing [87]. It is possible, by taking advantage of the low to moderate *h*^2^*s*, to select against the myopathies while continuing to improve biological performance through balanced breeding [81]. This is achievable due to the low genetic correlations between broiler production traits (i.e., body weight and breast yield) and myopathies. Published data from multiple analyses have shown that the correlations between the myopathies and the broiler production traits are moderate to low; this means it is possible to select birds with both optimal breeding values for breast yield and a reduced propensity for developing the myopathies [81]. In fact, an empirical study using so-called “high-generation broilers” indicated that through balanced breeding, a relative increase in breast yield of 1.2% can be achieved concurrently with a relative reduction of 9.2% in WB [86]. While the non-genetic factors are more influential when it comes to the development of breast myopathies, it is possible, as part of a holistic approach, to take advantage of their low to moderate *h*^2^*s* as a long-term strategy to reduce incidence.

### 3.3. New Methods

#### 3.3.1. The Impact of Genomics Selection

**Figure 19:**
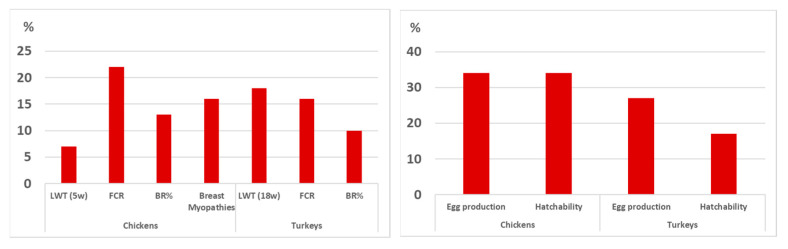
Improvements in breeding value prediction accuracy in production and reproductive traits from using genomics information in broilers and turkeys. **Left**: production traits. **Right**: reproduction traits. LWT—live weight; FCR—Feed conversion rate; BR%—Breast %.

The use of genomics information for predicting breeding values in broilers and turkeys is part of the routine operation of Aviagen’s breeding programs and has been widely reported, e.g., [88,89,90,91,92]. The availability of 50,000 Single Nucleotide Polymorphism (SNP) panels for broilers and turkeys allows important increases in the accuracy of breeding value prediction. Figure 19 shows that genomics contributes to extra selection accuracy in broilers and turkeys. For live and yield performance (LWT, FCR, Breast % (BR%) and breast myopathies in broilers), increases in the selection accuracy range from 7% to 22%, and for reproduction, genomics brings 17% to 34% extra selection accuracy. The lower, extra accuracy range for production traits is expected, as there is more performance information on selection candidates (including CT measurements for yield and meat quality); hence, the contribution from genomics is moderate. The relative increases in accuracy for reproductive traits are due to a more accurate estimation of Mendelian sampling thanks to the availability of genomic information. When young females are selected before starting the reproductive cycle, using pedigree-based EBVs, it is impossible to distinguish between full sibs, while when genotypes are available, this is feasible. The same benefits apply to male candidates as egg production and hatchability are sex-limited traits. The constant expansion of analytical tools to predict genomic EBVs and the increased computing power will allow for a continuous increase in the scale of genomics genetic evaluations, with tens of thousands of genotypes added every year.

The next frontier for increasing the accuracy of prediction is to utilize whole genome sequence data. It is becoming increasingly feasible, both in economic and practical terms, to obtain full sequence information on a large number of animals. However, using such a large dataset for genomic prediction is still challenging [93]. Although causative variants (or makers in very strong linkage disequilibrium) are expected to be contained in such a large dataset, the computational complexity of including all full genotypes in the analysis is prohibitive. In order to improve the signal-to-noise ratio, prior information can be used to filter out variants not associated with the traits of interest. Such information includes variants identified from Genome-Wide Association Studies and variants with a functional role. The latter highlights the importance of using updated genome annotation that is specific to the species of interest. International consortia, such as Functional Annotation of Animal Genomes [94,95] and Genotype-Tissue Expression [96,97], which incorporate the latest ‘omics data to bring the state of genome annotation on par with other species, enable poultry geneticists to significantly increase the resolution of mapping QTLs or potentially pinpoint the causative variants. Finally, in order to fully exploit sequence data, more flexible statistical models that explicitly account for different functional annotations are required.

#### 3.3.2. Gut Health and Gut Microbiome

The gut microbiome is a community of microbes within the intestinal tract; it plays a key role in developing and maintaining the immune system, gut function, digestion and protection against gut pathogens [98,99]. The microbiome has been a key area of research for decades, and now, with advances in technology and reduced costs, understanding the role of the microbiome is becoming more accessible within the livestock sector. The influence of the microbiome on host health is well documented, with exposure to different micro-organisms early in life having an influence on subsequent gut function and composition of the microbiome [100,101]. Research carried out in a wide range of animal species has shown that the composition of the microbiota is dynamic; there can be changes in the presence or abundance of species in relation to a range of factors such as host genetics, environmental conditions, nutrient intake, dietary formulation, and different health states [102,103,104,105,106]. In addition to its role in promoting host health, the composition and activity of the intestinal microbiome have been associated with feed efficiency and performance of the broiler [107,108,109]. Harnessing the power of the microbiome and understanding its relationship with animal performance is proving to be a new frontier within animal breeding. Data from pigs, cattle and laying hens have shown that not only are components of the microbiome heritable, but also the relative abundances of bacterial species within the microbiome account for a proportion of the phenotypic variance for traits such as feed conversion and methane emissions. Difford et al. (2018) [110] estimated that some members of the microbiome showed a *h*^2^ of 0.25 and accounted for around 0.15 of the variance for methane emissions in cattle.

Similarly, in pigs, Camarinha-Silva et al. (2017) [111] estimated that members of the microbiome showed a *h*^2^ up to 0.57 and accounted for 0.16, 0.21 and 0.28 of the phenotypic variance for feed intake, feed conversion and daily weight gain. In laying hens, it has been reported that 0.12–0.16 of the variance in traits such as body weight, daily feed intake and residual feed intake could be attributed to the microbiome [112]. This has led to the suggestion that animal breeding should include selection for the bacterial metagenome alongside that of the host to improve gut function and biological efficiency [113]. This is, of course, an exciting—albeit time-consuming and still costly—prospect; however, there are a number of considerations to be made. Firstly, are any of the bacterial species associated with important production traits a direct cause or simply the effect of other processes occurring in the intestinal tract? Secondly, as the microbiome is influenced heavily by factors such as dietary composition and environmental factors, would selection for a specific microbiome only be successful when the progeny of selection candidates are placed into an identical environment? Lastly, at what age would a sample for analysis be taken as the microbiome changes with bird age, and there may be a key age where the microbiome is more informative. These questions will likely be answered in the future once more data is collected, and then the practicality of selecting a specific microbiome composition can be determined.

When we consider the fact that the composition of the microbiome is heritable and accounts for a proportion of phenotypic variance, there is the potential to use microbiome data to improve the accuracy of selection. Within the high biosecurity farms where the pedigree birds are reared, there is a low level of bacterial challenge; however, the farms are not sterile. On these farms, management factors are kept homogeneous across farms to limit residual error; if the microbiome influences important production traits, it is only logical to want a similar bacterial landscape across pedigree farms so that any impact by the microbiome is uniform across farms. The uniformity of microbial landscapes across farms across the Aviagen turkey breeding program was carried out using 16S microbiome analysis of litter and fecal material from UK and US high hygiene (HH) pedigree farms and the low hygiene (LH) farms used for multi-environment selection. The data showed that there were very few significant differences between the pedigree farms samples at both the genus and species level, thus indicating a high level of uniformity of bacterial exposure across those farms.

**Figure 20:**
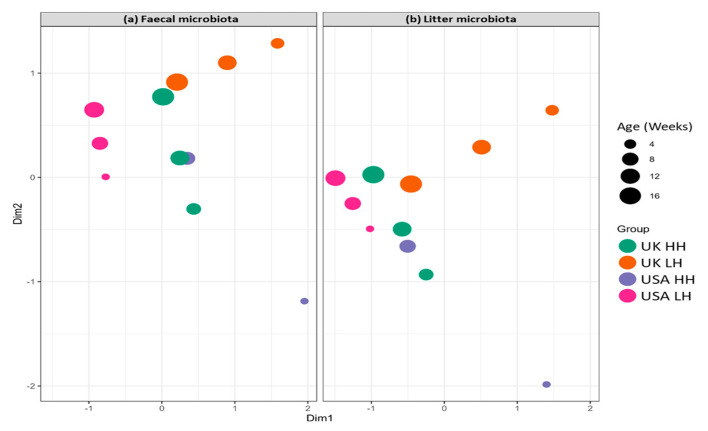
MDS plot of mean fecal (**a**) and litter (**b**) microbiota of key elite turkey pedigree lines at a range of different ages from both high hygiene (HH) pedigree farms and low hygiene (LH) farms in the UK and US breeding programs. The closer the markers, the more similar the microbiome in the samples. This plot reveals that the microbiome changes with age and also highlights that the LH environments are quite distinct from each other and the HH environments.

Conversely, the bacterial communities identified in LH farms were not only significantly different from the pedigree farms but also from each other; this highlights the vast diversity of bacterial species that can be present in the environment of the turkey. This approach offers the opportunity to monitor the bacterial communities present within the breeding environments and learn more about the dynamics of the microbiome over time and the impact of hygiene practices (Figure 20). Additionally, it will also be possible to obtain the microbiome of commercial farms from across the global industry to ensure that multi-environment selection provides exposure to relevant environmental bacteria to ensure the selection of traits associated with robustness, environmental adaptability and immunocompetence.

### 3.4. The Management of Trait Antagonisms

The key parameter controlling the extent of the genetic antagonism between traits in a breeding goal is their genetic correlation (GC): “The GC measures the extent to which the same genes control two traits. A favorable GC means that the genes controlling both traits have the same effect on each trait while an unfavorable or antagonistic correlation means that the effect is the opposite in each trait” [114]. The latter is called trait antagonism. It is often the case for combinations of traits impacting robustness, welfare, health, reproduction or adaptability with traits affecting production, yield or efficiency. It is seen in complex breeding programs with broad ranges of traits. This phenomenon is common and well-documented. Over thirty years ago, in broilers, increases in the incidence of ascites and skeletal abnormalities were reported “in birds intensively selected for growth rate” [115].

Overcoming trait antagonism is the basis and prerequisite of balanced breeding. Hill et al. (2016) [57] illustrated the principle of multi-trait genetic selection in the presence of trait antagonism in meat chicken breeding and demonstrated that when traits are included in a broad breeding goal and balanced selection is applied, the desired direction in each trait can be achieved. The way to handle these antagonisms is by estimating the genetic correlations between trait groups in the breeding goal (e.g., between biological performance and health and welfare or product quality) and ensuring that antagonistically related traits are improved simultaneously. This is possible when the antagonistic relationship between traits is mild to moderate.

#### 3.4.1. The Levels of Trait Antagonism

**Figure 21:**
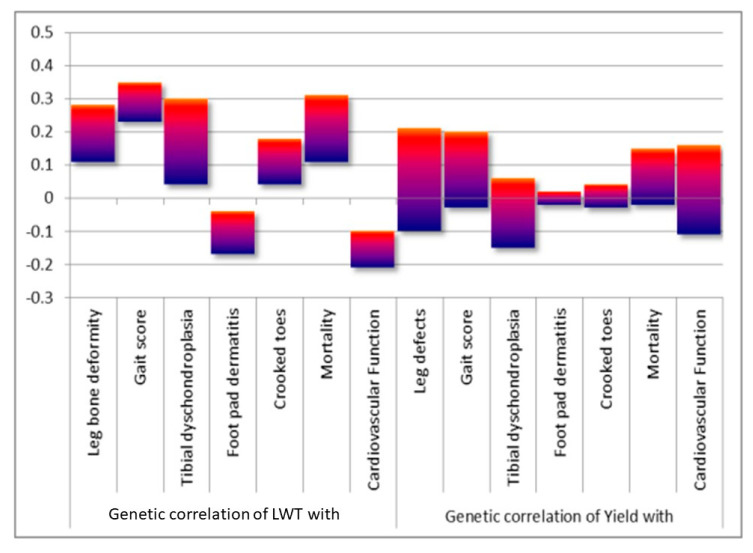
Broiler breeding program ranges of Genetic Correlations between Live Weight (LWT) and Breast Yield (BY%) with Leg Bone Deformities (%), Gait Score, Tibial Dyschondroplasia (%), Foot Pad Dermatitis (%), Crooked Toes (%), Mortality (%) and Oxygen Saturation levels in the blood (%). Source: [114].

Figure 21 shows that the GCs between body weight and yield with health and welfare traits like leg, joint and foot health, walking ability (gait), cardiovascular function, and livability vary from 0.33 to −0.2. These correlations are mild to moderate and, hence, are manageable in commercial breeding. This is confirmed by Hiemstra and Ten Napel (2013) [37], who found that the genetic correlations between health and welfare-related traits and production traits in broiler breeding programs were all in the range of −0.30 to +0.30, indicating that both groups of traits can be improved simultaneously.

#### 3.4.2. The Management of Antagonisms between Two Traits

**Figure 22:**
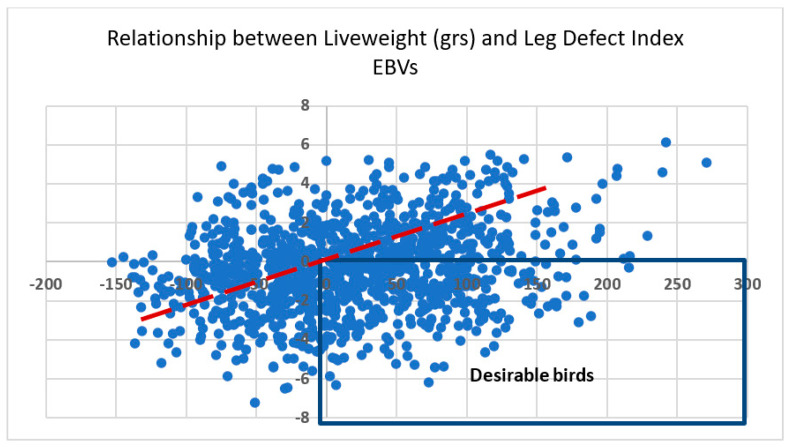
Estimated Breeding Values (EBVs) for leg strength (vertical axis) and live weight (LWT; horizontal axis) as deviations from the population mean.

With an example from the Aviagen broiler breeding program, the genetic antagonism between live weight (LWT) and leg defects is demonstrated. Figure 22 shows the estimated breeding values (EBVs) for a cohort of pedigree birds as deviations from the population mean. In this dataset, the GC between leg defects and LWT is 0.24, typical for the antagonism between health/welfare and production-related traits. The red-arrow trend line indicates that as we move further to the right, we will find birds with better LWT but more leg defects, while to the left, we find birds with lower LWT and fewer leg defects.

As explained by Hill et al. (2016) [57], dealing with this dilemma means that both traits must be included in the breeding goal, and birds should be selected that are good for both traits at the same time. The birds with breeding values in the right bottom box are the birds of this contemporary group with better EBVs than the population average for both traits at the same time.

**Figure 23:**
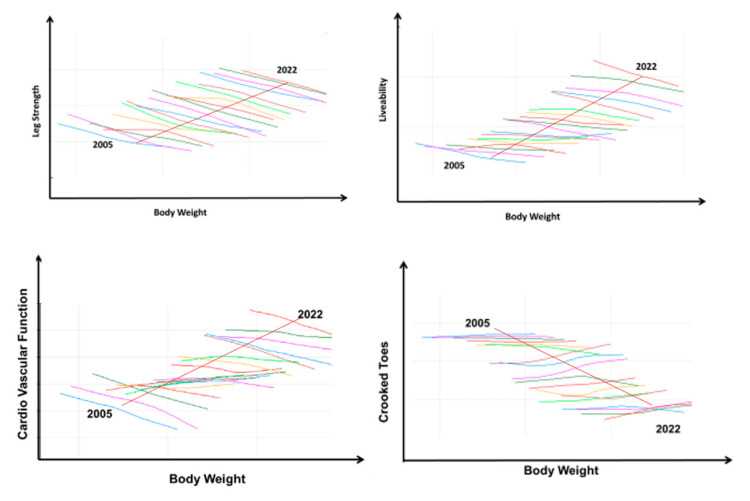
Long-term relationships between live body weight and leg strength (%), livability (%), oxygen saturation in the blood and crooked toes (%) in broilers. Each colored line represents the relationship between breeding values for each trait within a year. The broken arrow represents the joint direction of the average breeding value for each trait involved in the trade-off. Source: [116].

The example above can be expanded to other antagonisms and the simultaneous improvement of traits in the long term. Figure 23 illustrates that growth rate and leg strength have been improved simultaneously over the long term while the antagonistic genetic relationship between both traits holds within a year. The data, based on Aviagen breeding program data, describes the joint trajectory between bodyweight and oxygen saturation levels, livability, leg strength and crooked toes over 18 years from 2005 to 2022. Each colored line shows the relationship between the traits’ estimated breeding values (EBVs) for selection candidates hatched in a specific year. The broken arrow represents the joint direction of the average breeding value for each trait involved in the trade-off. The relationships between traits remain antagonistic within each year, but across time, there is a favorable trajectory for each trait because of simultaneous selection; that is, as BWT increases, cardiovascular function, livability and leg strength increase while crooked toes decrease.

## 4. Breeding for Sustainability: The Balance between Welfare and Environmental Impact

Balanced, holistic breeding contributes to the sustainability of poultry meat, as breeding improvements are permanent, cumulating generation on generation, and disseminated widely across the poultry supply chain. Incorporating novel recording and analytical approaches for predicting genetic values with higher accuracy enables both the genetic improvement of sustainability and welfare and the handling of trait trade-offs [114], it is also important to consider economic and societal forecasts, as breeding is a long-term exercise.

Feedback from the wider society influences how to balance health and welfare, and the environment impacts (short and long-term) breeding decisions made at the top of the breeding pyramid (Figure 1). Whitton et al. (2021) [117] distinguished two groups of countries depending on the relationship between Gross Domestic Product (GDP) and meat per capita consumption. One group of countries with low-income GPD shows higher meat consumption as GDP increases. In contrast, in countries with GDPs higher than USD 40,000, the link between GDP and meat consumption is not seen. In this group of countries, consumers may be more sensitive to environmental and ethical/animal welfare and health concerns.

### 4.1. Crossbreds for Different Market Segments

A broad portfolio of crossbreds will help satisfy the shifts and differences in requirements across countries and markets. In turkeys, next to the standard turkeys, slower-growing and colored turkey crossbreds are used for the Christmas market, e.g., [118]. In broilers, there has been a growing interest in meat arising from alternative production systems with various schemes targeting thresholds for growth rates lower than 50–58 g/day and/or specific requirements aimed to lead to higher (perceived) welfare in chicken production. Table 1 illustrates the growth rate, days to 2.5 kg live weight, FCR rate, yield, breast percentage, and the livability of standard and slow-growing crossbreeds in the Aviagen broiler portfolio.

**Figure 24:**
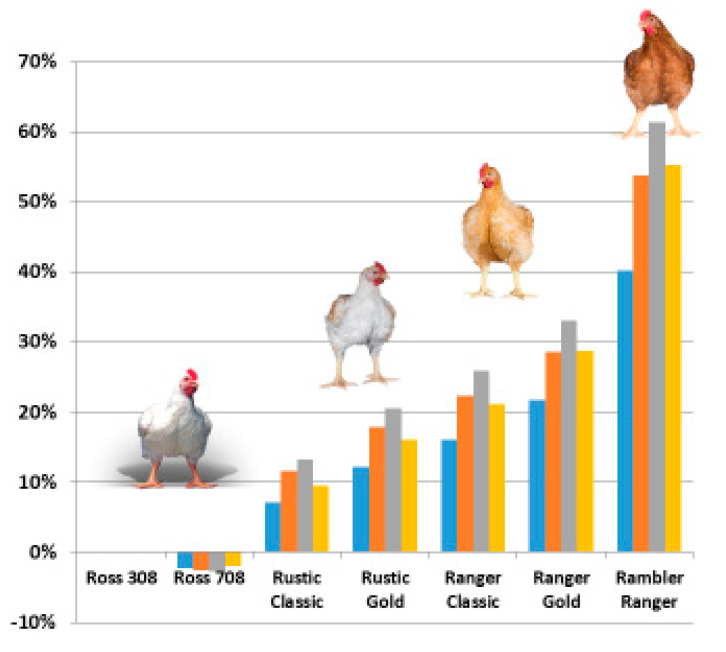
Environmental impact of different crossbreds. Global warming potential, Eutrophication potential, Acidification potential, and Primary energy use compared to Ross 308 taken as a baseline (0%). Blue—Global Warming Potential (GWP, Carbon Footprint, CO_2_ eq); Orange—Eutrophication Potential (kg PO_4_ eq); Grey—Acidification Potential (kg SO_2_ eq); Yellow—Primary Energy Use (MI). eq—equivalent.

Ross 308 and Ross 708 are globally established commercial broiler crossbreds; Rowan Range broiler types are slower-growing crossbreds (eu.aviagen.com/brands/rowan-range). The Rowan Range genotypes fall under the requirements of accreditation schemes like Better Chicken Commitment (BCC) [119], ‘Kip van Morgen’ [120] and ‘Beter Leven’ [121] in the Netherlands, ‘RSPCA Assured’ (RSPCA) [122] and Red Tractor Enhanced Welfare [123] in the UK, and ‘Für mehr Tierschutz’ [124] in Germany. These systems address the concerns of consumers in higher-income countries about meat production systems, in particular animal welfare, including traceability [3]. It is important to note the very significant differences in performance between conventional and slow-growing crossbreds, for example, FCR of 0.62 g feed/kg live weight (Ross 308/708 vs. Rambler Ranger) and Breast % (BR%) of 8.9% (Ross 708 vs. Rambler Ranger). These differences represent about 30 and 40 years of selection for FCR and BR%, respectively. On the other hand, the greatest difference in livability between conventional and slow-growing crossbreds in Figure 24 is only around 2% (e.g., Ross 308 vs. Rambler Ranger). This very narrow difference is due to the emphasis of selection on welfare and livability applied to the whole portfolio of crossbreds.

### 4.2. Environmental Impact of Different Crossbreds

While slower-growing breeds are associated with better welfare outcomes, they have additional environmental costs. Environmental sustainability is another area of growing interest “as consumers and producers become more aware of the increasing global population along with the increased need to make better use of available resources” [72].

Using a Life Cycle Assessment (LCA) tool developed by Cranfield and Newcastle University (Poultry LCA Model Version 1.0) [125,126], a breeding company can calculate the environmental impact of its broiler crossbreds on a regular basis, e.g., [114]. Figure 24 shows that the more biologically efficient crossbreds have the lowest environmental impact in terms of pollutant emissions and other outputs in LCA. As average daily gain and breast yields increase, global warming potential (GWP) decreases linearly—the opposite trend is seen for FCR. These results are consistent with Leinonen et al.’s (2012) [126] findings: a free range and an organic production system had a predicted higher GWP of 16% and 28%, respectively, over a standard production system. These findings are in line with Herrero et al. (2013) [127], which conclude that feed efficiency is a key driver of productivity, resource use and greenhouse gas emissions.

The overall conclusion is that the environmental impact of crossbreds with lower biological efficiency is 30–40% higher compared to conventional genotypes. FCR differences were also the main driver for environmental impact differences in LCA research in turkeys [128,129] and in a study comparing male and female turkeys grown in two ventilation systems [130,131,132].

### 4.3. Outcomes at the Farm Level

The suitability of a crossbred to a production system or market segment will depend not only on biological performance but also on consumer preference, product price and other product attributes, including any perceived compromise between performance, welfare and environmental impact [114].

With interest in welfare increasing, the value of robust welfare measurements on the bird is growing. Canadian government data (Figure 9 and Figure 12) show that improvements in genetics over time are translated into improvements at the level of commercial producers. Apart from genetic potential, management also plays a key role in the welfare outcome of the bird.

In a Dutch multi-stakeholder project, Greenwell [133,134] examined the environmental, welfare and economic aspects of farming concepts used in the Netherlands. The three major farming systems compared were the conventional system (fast-growing birds, standard conditions), Dutch Retail (broilers with maximum 50-g growth per day, farming conditions close to Better Chicken Commitment) and Beter Leven 1 star (maximum growth per day of 45 g, additional management requirements such as lower stocking density, environmental enrichment, early feeding of day-old chicks). Animal-based welfare indicators assess the outcome of the bird, and resource-based indicators consider the inputs, for instance, breed or environmental enrichment. In the welfare aspect of the study, a Total Welfare Score (TWS) was developed with five animal-based and three resource-based indicators [135]. These criteria were used to calculate welfare scores for farms operating the different concepts [136].

**Figure 25:**
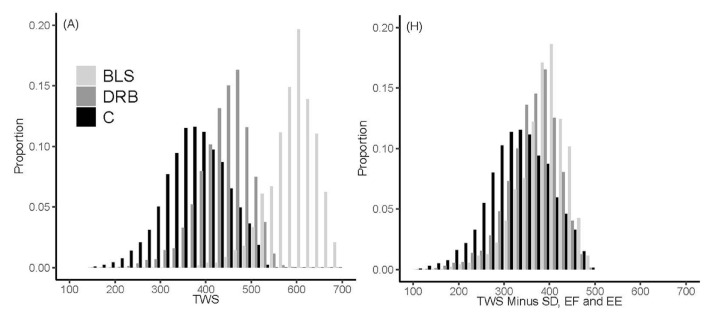
Histograms of the distribution of the Total Welfare Score (TWS) for flocks of the three production systems (**Left**), including eight animal- and resource-based welfare indicators (**left**) and five animal-based welfare indicators (**Right**; TWS—resource-based indicators). The animal-based indicators were mortality, footpad, hockburn, breast irritation and scratches; the resource-based indicators were: SD (stocking density), EF (early feeding), and EE (environmental enrichment). DRB—Dutch Retail Broiler; C—Conventional; BLS—Beter Leven System (Source: [136], Figure 1A,H).

Figure 25 shows that, when all welfare indicators were included, there were three distinct but overlapping distributions, with the Conventional (no resource-based scores) being the lowest, followed by the Dutch Retail (one resource-based score) and then the Beter Leven 1 star (three resource-based scores). However, when the resource-based indicators were removed, and only the animal-based indicators were considered, the variation between the concepts was vastly reduced, with a very large overlap between the three systems.

This clearly shows the importance of management, as conventional birds can be farmed with as good or better animal-based welfare scores than Beter Leven 1 star, depending on management. To assist producers in the field, a multi-stakeholder initiative, including industry representatives and scientists known as the International Poultry Welfare Alliance, has recently published a resource guide on animal-based Key Welfare Indicators—a reference to help those managing and caring for poultry to understand key welfare indicators and how they can be used to improve welfare outcomes [137,138].

## 5. A Future Outlook

The OECD/FAO (2023) [3] predicts that the global population will grow by 11% from 7.9 billion in 2022 to 8.6 billion in 2032. At the same time, global meat production is expected to expand by 15% to 2032, with poultry meat expected to account for 48% of the increase in the coming decade. A positive outlook is that in the next decade, the meat sector’s greenhouse gas emissions (GHG) are expected to rise by 7.6%, which is lower than the projected 12.8% growth in agricultural output. This is clearly linked to poultry production being more efficient and less resource-intensive, making it a more sustainable choice for meat [3]. Our estimates indicate that poultry breeding has contributed to the 50% lower carbon footprint compared to the broiler in 1970. With the continued investments and expected improvements in biological efficiency, the broiler in 2030 is predicted to generate a 15% smaller footprint than the broiler of today. These predictions are very much in line with predictions by the OECD-FAO for the trajectory of poultry meat expansion and the concomitant reduction of carbon footprint contribution from the meat sector expected in the next decade.

At the same time, 90% of the total increase in meat production and 73% of the increase in poultry meat will come from developing economies [139]. This clearly indicates that current and future commercial poultry crossbreds will need to adapt to a very wide range of geographies and production systems, highlighting that selection for robustness and environmental adaptability will be of key importance. As populations move away from rural communities and into towns and cities, the links with agriculture are reduced; the understanding of how food is produced is decreased as it no longer forms a part of everyday life for most of the population. Increasingly, the consumer wants to choose what to buy and is becoming concerned about how their food is produced [72]. Consumers in high-income countries want to choose from various options, including diverse poultry offerings. One way to fulfill this trend is to offer a broad portfolio, including colored and slower-growing options or labels certifying different farming options for which different breeds may be the preferred or prescribed option. Due to the movement from a producer to a consumer market, the buying power of the consumer has a greater influence on what food is produced. In particular, the influences of the retail and food service sectors and animal welfare considerations on consumer preferences have been growing during the last decade.

The role of animal protein in the diet is also challenged by food system discussions at global policy levels for environmental impact or health reasons, and, on the other hand, confirmed by key scientists, e.g., [140]. Animal protein-poor diets, like the EAT-Lancet diet, focus on metrics such as calories or protein while other key nutrients such as vitamins, minerals and bioactive compounds are neglected, e.g., [141,142,143]. In addition, the “sustainable” options per kg or per kcal are often poor nutrient sources, and diets low in animal-based foods are usually less nutritionally robust [144]. Animal proteins contribute to cognitive development and the prevention of runting and stunting [140].

It is expected that the reduction of the use of antibiotics will continue in animal production, meaning that the robustness of the birds will continue to be an important characteristic of poultry breeds.

As Avendaño et al. (2017) [114] predicted, breeding should continue to expand “breeding goals with the ability to change and adapt as a response to current and future changes in global trends”, i.e., more balanced progress across more traits. In addition, a broad genotype portfolio will provide a broiler and turkey meat production spectrum that can address global demand in an environmentally sustainable way and serve emerging niche markets (such as free range or organic). This requires (a) large genetic pools that are suitable to fulfill current and future needs in the market and society and (b) continued investment in research and development to provide for the genetic basis of novel traits and emerging genetic correlations and weighings in new and existing traits and trait areas in the breeding goals of the various lines. Novel recording and selection techniques, as well as an improved understanding of behavioral and biological functions like gut health. The obtained knowledge can contribute to improving the genetic lines, contributing to the range of conventional and specialty crossbreeds of the future. Clearly, the focus from the primary breeder will be to offer the genetic potential suitable for all market segments and future options while fulfilling sustainability requirements from economic, biological, welfare and environmental considerations. The long-term structure of breeding involves a long process of anticipation and development: appreciation of future needs, adaptation of the pedigree genotypes and then at least four years of multiplication from pedigree to bird on the market. The genetic potential at the pedigree level will be transferred to the market in around four years—these birds are currently being selected and entering the trajectory of multiplication. Furthermore, regardless of the future trajectories of market and consumer requirements, poultry breeding will still rely intensively on research, development and the adoption of technologies and techniques that can predict breeding values as accurately as possible and will be characterized by the dynamic development of commercial crossbreeds to satisfy the wide range of market needs.

## 6. Conclusions

Since the 1950s, animal breeding has been very successful at increasing livestock productivity potential. Since the 1970s, poultry breeding goals have increasingly broadened from a narrow focus on productivity to include a wide range of aspects improving at the same time productivity, efficiency, environmental impact, animal health and welfare, food quality and safety and genetic diversity with demonstrably improved welfare, environmental impact and affordability of poultry meat. This expansion in breeding goals has been underpinned by an intense research process, development and adoption of novel selection techniques, which is expected to continue in the future. With the continuous global expansion of poultry meat production and consumption, the portfolio of commercial crossbreeds is expected to keep expanding to satisfy a wide range of market and consumer preferences. With welfare, environmental impact and cost of poultry production improving over time, poultry remains in a good position to contribute to lean, healthy animal food availability worldwide.

## Figures and Tables

**Table 1:** Characteristics of crossbreds in the Aviagen portfolio at 2.5 kg live weight. Average daily gain (ADG), days to 2.5 kg, feed conversion rate (FCR), % eviscerated yield and breast yield, and % livability.

Crossbred	ADG	Days to 2.5kg	FCR 2.5kg	% Evis	% Breast	% Liveability
Ross 308	66.3	37.7	1.53	73.4	25.3	96.5
Ross 708	64.1	39.0	1.53	74.5	27.0	97.0
Rustic Classic	57.0	43.9	1.68	72.7	24.4	97.5
Rustic Gold	52.0	48.1	1.75	71.9	23.2	98.0
Ranger Classic	48.7	51.3	1.80	71.3	22.3	98.1
Ranger Gold	44.0	56.8	1.87	70.5	20.9	98.3
Rambler Ranger	33.0	76.0	2.15	69.3	18.1	98.5

## Data Availability

Not applicable.
